# Gaps and Challenges in Harnessing the Benefits and Opportunities of Indigenous Certification for a Sustainable Communal Commercial Lobster Fishery

**DOI:** 10.1007/s00267-023-01852-7

**Published:** 2023-07-20

**Authors:** Isa Elegbede, Melanie Zurba, Ahmad Hameed, Chelsey Campbell

**Affiliations:** 1 Department of Environmental Planning, Brandenburg University of Technology, Cottbus, Germany; 2 School for Resource and Environmental Studies, Dalhousie University, Halifax, NS Canada; 3 Department of Fisheries, Lagos State University, Ojo, Lagos Nigeria; 4 The Confederacy of Mainland Mi’kmaq Mi’kmaw Conservation Group, Halifax, NS Canada

**Keywords:** Indigenous certification, Policy research, Lobster seafood and fisheries, Boundary work, Sustainability, Participatory research

## Abstract

The Marshall Decision of Canada’s Supreme Court inspired the Mi’kmaq in the 1700s regarding recognizing fishing rights to the Mi’kmaq communities. Despite this recognition, the Mi’kmaq communities did not have access to commercial fisheries due to the denial of absolute recognition of territories and rights and underrepresentation and participation in resource allocation, governance, and decision-making processes. A potential approach to these issues is the development of third-party Indigenous community-based sustainability certification standards for the American lobster (*Homarus americanus*) commercial fishery of Nova Scotia by Mi’kmaq communities. An Indigenous certification is a market-based tool that focuses on a holistic approach to the sustainability of the resource, followed by independent accreditations and standards. This study identifies the gaps, challenges, and opportunities of Indigenous-based certifications for the American lobster commercial fishery. We adopt a participatory approach to conventional policy analysis and perform a secondary analysis of existing legal and scientific resources to glean valuable information for supporting the establishment of an Indigenous certification for the American lobster. Certification could provide benefits such as increased control over fisheries management, governance, rights, and socioeconomic interest, building capacity for Mi’kmaq communities, and improving stakeholder relationships. However, there are issues with the entry points of certification for Indigenous peoples related primarily to the dominant actors in accreditation. This study will support further research and engagement of the Mi’kmaq people toward developing an Indigenous certification scheme.

## Introduction

The Mi’kmaq (pronounced as Meeg-maw and literarily meaning “The Family”) are Indigenous peoples of the Maritime Provinces of Canada, the Gaspé region of Québec and the northeastern region of Maine in the United States (Berneshawi, 1997; Fox, 2006; Trenholm et al., 2019). They are northeastern Algonquian-speaking people organized into bands dictated through the Indian Act with a deep cultural connection with the stewardship responsibility to maintain terrestrial and aquatic resources based on the concept of Netukulimk^1^ (Berneshawi, 1997). The American lobster (*Homarus americanus*) is an essential fishery to the Mi’kmaq due to its availability year-round, popularity among the people, and nutritional potential, which commanded high market value and has been the economic backbone of the community since the 1800s. This fact conforms with the fact that it is cited as one of the most dominant coastal fisheries in Atlantic Canada, providing more than 50% of the global supply of lobster (Bond, 2017). Commercial lobster fisheries represent approximately one-third of commercial seafood exports from the Atlantic region, with an economic value of approximately CAD 2.1 billion in 2017 (DFO, 2018; DFO, 2021; Dave & Routray, 2018; Castañeda et al., 2020).

Presently, the lobster fishery in Atlantic Canada is mainly controlled and regulated by the government through the Department of Fisheries and Oceans (DFO) without extending compliance and enforcement powers to Mi’kmaq communities (DFO, 2019). However, this situation contradicts the Marshall Decision that recognized that the Mi’kmaq people have the right to fully access and explore lobster fishery resources for communal commercial benefits (McMillan & Prosper, 2016; Poliandri, 2003). However, in November 1999, in the decision of Marshall II, the Supreme Court specified that the federal and provincial governments (within their respective jurisdictions) have the power to regulate the exercise of treaty rights (Borrows, 2000, 2001; Saunders, 2000; Ladner, 2009). However, this power is subject to constitutional requirements that limitations on exercising the right must be justified. This situation includes limiting treaty rights on conservation grounds, and conservation is largely understood as being the most crucial reason that the government could invoke to limit treaty rights. However, other reasons could include ensuring the economic viability of the existing non-Indigenous fishery (Wiber & Barnett, 2021; McMillan & Prosper, 2016). In line with understanding the relevance of the lobster fishery to the Mi’kmaq, this policy research focuses on the interest of Mi’kmaq fishers with the prospect of exploring new economic opportunities within communal commercial lobster fisheries through an Indigenous certification system. The study engages a relationship between academics and partners from the Mi’kmaq Conservation Group (MCG), an affiliate of the Confederacy of Mainland Mi’kmaq (CMM) of NS. The MCG advisory board members and staff determined the questions driving this policy research as a potential step toward developing an Indigenous certifications program, which could be decided as a third party or as a supplement to an existing independent certification program. This Indigenous certification could be a platform to sustainably manage Mi’kmaq lobster fisheries and other endowment resources to benefit Mi’kmaq communities and future generations. The research further focuses on the potential of the Indigenous-based lobster fishery, which has aspects that are conveyable to other Indigenous or traditional fishing types worldwide. Specifically, the research seeks to identify challenges, gaps, and opportunities in harnessing the benefits of this kind of certification programme for Mi’kmaq communities in the entire Atlantic region.

## Background

This article distinguishes between communal commercial fisheries and rights-based fisheries. While certifications related to the commercial fishery, it is still important to situate this study within the broader context of the different types of fisheries that Mi’mkaq can be involved in.

### The Communal Commercial Fishery

Prior to the arrival of the Europeans, Mi’kmaq communities participated in lobster fisheries for two reasons: culturally and commercially along the inshore. In the past, the Mi’kmaq people lived in the coastal areas of eastern Canada long before the seventeenth-century advent of Europeans. The Mi’kmaq were nomadic people who were divided into family-based clan groups or Bands. They had a rich culture that drew on the resources and environment of their surroundings (Wicken, 1994, 2002; Milley & Charles, 2001). The Mi’kmaq people have historically relied on the sea for transportation, trade, and sustenance, similar to other coastal populations worldwide. The fishery significantly influenced the communities’ yearly movement patterns. A migratory life cycle that included hunting, fishing, trade, and obtaining everything the earth had to give included the fishery as a significant component. More than 90% of the available food in the precontact era, according to estimates, came from ocean resources. It has played such a significant role in the Mi’kmaq way of life that it has intricately woven into their worldviews, as well as those of the Maliseet and Passamaquoddy, two nearby First Nations. This way of existence developed a cyclical social and political culture that drew from these natural rhythms. As one might anticipate, the significant reliance on wild resources for food led to the formation of spiritual perceptions of the universe, mythology to explain natural occurrences, and social structures and codes of behavior to specify proper harvesting methods (Milley & Charles, 2001). Through a series of treaties, first with the French and then the English, the Mi’kmaq expanded their domestic legal systems once Europeans arrived on the beaches of Mi’kmaq. Between 1725 and 1794, Britain and France negotiated treaties (Wicken, 2002).

The Mi’kmaq way of life needed to be protected against the British’s superior political and military might. Hence, the treaties were made mainly for peace and friendship. They did not give up any Mi’kmaq lands or resources. A royal proclamation was made in 1763 that secured the Mi’kmaq people’s unimpeded use of hunting grounds and acknowledged the Mi’kmaq people as a nation (Milley & Charles, 2001).

Nonetheless, because of the peace and friendship treaties, the European colonial expansion into the Atlantic provinces continued to the point that the descendants of Europeans considerably outnumbered the Mi’kmaq. Government rules and policies governing Aboriginal people and the contemporaneous emergence of fisheries laws and policies governing the management of non-native commercial fishing both impeded Mi’kmaq’s access to natural resources during this time. By a practice known as centralization, Mi’kmaq people were relocated to reserved areas and prohibited from using their language in formal educational settings (Milley & Charles, 2001).

Individuals or families could only grow economically in the contemporary North American economy if they or their children left the reserve to look for work and pursue an education outside of the confines of their cultural background. Due to the extremely high unemployment rates in Mi’kmaq communities today, this need for social support is a defining characteristic. In many cases, the unemployment rate in Mi’kmaq communities is more than 80% (Wicken, 2002). Due to these circumstances, the relationship between Mi’kmaq Bands grew increasingly dependent on the current political climate rather than the customary Sante Mawiomi. Additionally, the federal and provincial governments’ management regimes supplanted Mi’kmaq management traditions.

### The Sparrow Decision

In a case involving a native fisher from British Columbia on Canada’s west coast, the Supreme Court of Canada rendered a historic decision in 1990 that had a significant effect on Mi’kmaq access to and participation in the fisheries (Kenny & Parenteau, 2014). The Supreme Court of Canada recognized the rights of Indigenous people to fish for food, social reasons, and ceremonial purposes in the Sparrow judgment. According to the Act (1982), Aboriginal people’s rights to the fishery have priority over other uses of the fishery, including commercial fishing, but these rights are subject to overriding considerations such as conservation (Doyle-Bedwell & Cohen, 2001). Additionally, it stipulated that whenever the rights of Indigenous groups would be impacted, the Canadian government must consult with those groups. Following the Sparrow ruling, the Mi’kmaq expressed a new interest in the fisheries and established Mi’kmaq’s sovereignty over their fishing operations. The Native Fisheries Strategy was the Canadian government’s response (AFS).

By agreements that established a federal licensing framework as a control mechanism for Mi’kmaq food fishing activity, the federal government made financial assistance for employment and economic development available to Mi’kmaq Bands under the AFS (Kulchyski, 1994). Within each of the Mi’kmaq villages, this became a point of conflict. Band Councils had the chance to help their communities financially by securing funding that would provide much-needed jobs. However, these agreements also reduced Mi’kmaq’s control over their harvesting practices. Throughout the 1990s, numerous villages entered into agreements with the government, further separating Bands from one another and displacing the Mi’kmaq communities from the community-based administration system of the past. The need to build management decision-making procedures decreased as bands’ reliance on the government to provide fishing permits increased (Ladner, 2009; Tobin, 1999).

Despite government regulation, a small number of Mi’kmaq communities continued to fish. These communities strongly supported a Mi’kmaq fishing management system. With this political motivation, the bands that had declined to sign the AFS agreements collaborated with the bands that had signed the AFS agreements to create regional fishery management programs. The Mi’kmaki Native Fishing Service was created in Eskasoni, Cape Breton Island, as the first of these (Wiber & Milley, 2007; Prosper et al., 2011; Harris & Millerd, 2010). To organize the operations of the fishery personnel employed under AFS agreements, the MAFS collaborated with several Mi’kmaq villages in Cape Breton with financial assistance from the AFS. The tremendous expertise gained by the MAFS, later renamed the Eskasoni Fish and Wildlife Commission, in implementing federal fishery regulations and programs at the local level provided the groundwork for new methods of community-based management in Mi’kmaq. The Mi’kmaq Fish and Wildlife Commission, which the Assembly of Nova Scotia Chiefs founded in 1995 to oversee natural resource activities on behalf of the Mi’kmaq and their institutions in Nova Scotia, was created as a result of the MAFS’s efforts (Milley & Charles,^ 2001^).

The MFWC had something in common with comparable Mi’kmaq fishery management programs in other parts of Atlantic Canada, such as the Fishery Division of the Union of New Brunswick Indians, in that it primarily relied on governmental funding sources, especially DFO and the AFS program (Milley & Charles, 2001). The chiefs and employees of the local Mi’kmaq management organization faced a conundrum. The Fisheries Act’s management control systems were regarded as conflicting with efforts to build an independent fishery management capability inside Mi’kmaq communities, which would compromise financing (Milley & Charles, 2001). However, if the groups tried to guarantee that the federal management system was implemented, it would be seen as conflicting with the objectives of the Mi’kmaq communities and would weaken political support. The reliance of Mi’kmaq villages on outside money made it clear that the AFS significantly influenced the development of Mi’kmaq management systems (Prosper et al., 2011; Milley & Charles, 2001).

### The Marshall Decision and its Repercussions

The Mi’kmaq harvester Donald Marshall Jr. was accused of engaging in illegal commercial fishing and charged under the *Fisheries Act*. This case has subsequently been refereed to as the Marshall case. Marshall’s legal defence had received backing from several Chiefs, who argued that the Mi’kmaq have a treaty right to fish for commercial purposes (Coates, 2000; Wicken, 2007; Wildsmith, 2001). The case also resulted in the establishment of Mi’kmaq Fish and Wildlife Commission (MFWC) by the Assembly of Nova Scotia Chiefs.

The chiefs believed it was essential to establish a Mi’kmaq (community) management system to guarantee a smooth transition for the Mi’kmaq into the commercial fisheries and prevent confusion regarding management priorities in advance of a decision in Marshall’s favor by the Nova Scotia courts (Prosper et al., 2011; Milley & Charles, 2001). Over a period of five years, the Marshall case was transferred from one court to another and eventually referred to the Supreme Court of Canada. The MFWC worked with individual communities to form local fish and wildlife management committees and establish local plans reflecting traditional values and contemporary aspirations (King, 2011; Wiber & Milley, 2007). In the Marshall case, the defendant won the Supreme Court of Canada’s (SCC) decision in September 1999. The commercial fishery’s Mi’kmaq treaty rights were acknowledged by the court (Fox, 2006; Prosper et al., 2011; Milley and Charles, 2001). In the aftermath of the decision, the Mi’kmaq communities felt vindicated while non-native fisheries experienced a period of uncertainty and were deeply concerned (Milley & Charles, 2001). The news was welcomed by the Mi’kmaq Chiefs and they responded swiftly by convening coordinated gatherings of Mi’kmaq, Maliseet, and Passamaquoddy Chiefs from across Atlantic Canada under the Atlantic Policy Congress of First Nations Chiefs, a policy advisory group founded by the Chiefs in 1992. (Orr & Weir,^ 2013^; Milley & Charles, 2001).

It was decided to avoid a fragmented response to the Marshall decision in favour of a unified and coordinated one (Fox, 2006; Wicken, 2002; Milley & Charles, 2001). A technical committee of fishery staff from several organizations was put together to support the Chiefs. This committee was mandated to help negotiations between the First Nations and the Canadian federal government on a seamless transition of the “Marshall fishery” between nations. Additionally, a negotiator was designated to represent communities in talks with the federal government (Milley & Charles, 2001).

One of the first actions the Mi’kmaq chiefs in Nova Scotia took following the Marshall judgment was establishing formal communication with non-native fishing organizations to allay concerns and advance their shared interest in a sustainable fishery (Fox, 2006; Milley & Charles, 2001). Interestingly, following strong reaction to the decision, the SCC issued clarification to the original decision in the same year (Isaac, 2001). This clarification explained the earlier decision and elaborated limited conditions under which Treaty Rights to fish could be curtailed (e.g., conservation). This has sparked ongoing debate to implement shared local administration with non-native partners. Many of these endeavours were successful, with organizations of inshore fishermen engaged in local initiatives to build community-based management (Coates, 2000; Wicken, 2007; Milley & Charles, 2001). Conversely, several organizations voiced their concerns regarding the decision and were unwilling to collaborate on local and community based management initiatives. Many of these organizations advocated for the SCC to rehear the Marshall case on grounds of profit and employment loss for fishermen. (Coates, 2000; Milley & Charles, 2001).

James MacKenzie was the sole negotiator hired by the Canadian government in 1999 to negotiate access agreements with Bands. Negotiated agreements had to be attached to non-native fishermen’s voluntary buybacks and the adoption of federal licensing regulations after substantial lobbying and pressure from non-native groups. (Coates, 2000; Milley & Charles, 2001). According to Coates (2000), the state can still limit Mi’kmaq treaty rights, primarily for environmental reasons under the Marshall decision and its subsequently issued clarification (Fox,^ 2006^; Prosper et al., 2011). Between the Marshall judgments, the Department of Fisheries and Oceans of Canada seized Mi’kmaq fishermen’s fishing equipment, including lobster traps (Johansen, 2001). Approximately a decade before the Marshall case, the SCC allowed the Mi’kmaq community their right to engage in the fishing for Food, Social, and Ceremonial FSC purposes. Under the Sparrow court ruling in 1990, legal clarification justified the infringements on Aboriginal rights based on “conservation and resource management need” (Giles et al., 2016; Gauvreau et al., 2017).

However, since Indigenous peoples are historically conservationists, the Mi’kmaq recognizes the term “conservation” as a connection to fish stocks with extended socioeconomic, political, spiritual, and cultural values tied to the community in line with the word *Netukulimk* (King, 2011). It is imperative for nation states to fully confirm the rights of the Indigenous peoples following legal rulings. Before colonisation, SCC also recognized the 1760 and 1761 Treaties of Peace and Friendship (TPF) without surrender of land rights, known as the Simon decision in 1986 (Cruddas, 2019; Davis & Jentoft, 2001). A series of negotiations occurred between the Mi’kmaq and Britain around the latter end of the imperial struggles between Britain and France. These events led to the signing of the TPF. Most SCC-established decisions have been interlinked with the existing 18th-century treaties in Canada (Davis & Jentoft, 2001; Castañeda et al., 2020).

### Post-Marshall: Development of Mi’kmaq Commercial Fisheries

The repercussions of the Marshall decision were far-reaching and resulted in the establishment of a nascent Mi’kmaq commercial fisheries focused on the right to moderate livelihood enshrined in the decision, after multiple negotiations and agreements with the federal government (Wiber & Miley, 2007). Several programs were introduced by the government through DFO to stabilise the post-Marshall fisheries landscape. These included buying back licences from non-native fishers and hiring non-native mentors for First Nations fishermen who were newly engaging in the sector (Wiber & Miley,^ 2007^). While some initiatives resulted in helping First Nations engage in commercial economic activities around fisheries and improve livelihoods, many were detrimental towards the development and success of Fish Nations moderate livelihoods post-Marshall (Wiber & Miley, 2007).

Mi’kmaq First Nations engage in commercial fisheries owned and operated by the community, and the regulated catch was sold for a profit. As a result of Mi’kmaq attempts to express their moderate livelihood right, the Supreme Court issued an action in 2003 that allowed the DFO to regulate these fisheries for conservation and good governance. As a result, commercial fishery allocation and access regulations are invoked, specifically for input controls (i.e., controlled seasons and gear restrictions) typically managed by DFO. Each of the communities is given some amount of communal commercial licences to operate. Communal commercial licences allow the community to decide who fishes those licences (Davis & Jentoft, 2001; Castañeda et al., 2020; McMillan & Prosper, 2016). Regular commercial licences are also available to the Mi’kmaq, and some Mi’kmaq people hold one. These municipal business permits were granted by the Native Communal Fishing Licences Rules (Wiber & Milley, 2007; Harris & Millerd, 2010). The relinquishment and replacement of Communal Commercial Access mechanism, created in collaboration with Indigenous partners and DFO, was used to handle requests from the First Nations to permanently transfer quotas or access (Stiegman, 2011; Castillo et al., 2021). This procedure has already been employed a few times. Regular fishery regulations typically apply to temporary transfers. The DFO establishes requirements for commercial licences at the industry level. The Aboriginal Communal Fishing Licence Regulations apply to Indigenous communities participating in the commercial fishery. However, they also contain the exact legal requirements (such as conservation measures, gear marking requirements, and reporting requirements) as all other commercial fishing licences (Theriault et al., 2014). Crab, lobster, scallops, sea urchins, groundfish, shrimp, swordfish, tuna, elver, clams, alewife/gaspereau, herring, and other seafood are among the items for which the Mi’kmaq people hold communal commercial permits.

Following the Marshall Decision, the DFO launched several initiatives inside existing government-driven institutions for greater Indigenous access to fisheries resources. This circumstance resulted in structural changes. DFO invested in transferring licences, vessels, gear, and fisheries infrastructure as part of the Marshall Response Initiative, which ran from 2000 to 2007 (McMillan & Prosper, 2016). Individual agreements with each First Nation are at the heart of this DFO initiative, which aims to increase Indigenous peoples’ access to fishing.

The Marshall Response Initiative was implemented to follow the Marshall 1 decision and prioritise treaty rights. While negotiating with the Mi’kmaq in Marshall Response Initiative negotiations, DFO negotiators made it clear that commercial agreements were not an implementation of a moderate livelihood treaty right. Some Mi’kmaq Nations might not have accepted these agreements if they thought they were defining how their rights would be practised and limited (Bailey & Charles, 2023).

The Mi’kmaq are treated as any other stakeholder under the current commercial licensing regime, and their collective interest in fisheries management for commercial benefit is not accommodated. The DFO licences commercial communal commercial fisheries. In contrast, the moderate livelihood fishery relates to the rights-based fishery. The rights-based fisheryIt connects to the Marshall decision, allowing the Mikmaq to assert their right to develop Indigenous-based certification without the DFO issuing licence. The Marshall decision recognizes the right to develop an Indigenous fishery outside of DFO’s system. This, in turn, can facilitate the development of an Indigenous fishery in their community and, as a result, contribute to their economy (Wieland et al.,^ 2016^). Regarding the commercial lobster fishery, Canada still needs to meet the requirements of Sparrow to consult Mi’kmaq in making decisions about implementing a moderate livelihood fishery. It is pertinent to note that sparrow requirements are limited to FSC, not commercial fisheries. However, the decision of the government to ignore and subsequently not give support to nations that chose to develop a moderate livelihood fishery might have contributed to their failure.

### Indigenous-Based Sustainable Fisheries Certification

Sustainability certification is a series of coordinated activities producing a product or service, which includes standard-setting, auditing, and complying with the standards. After that, products are given labels or logos validated by establishing institutions that guarantee quality control (Van der Ven, 2019). Certification can be governmental, nongovernmental, or private enterprises. Similarly, they could be market-based or nonmarket-based, and not all accreditation is attached to labels or logos (Vandergeest et al., 2015; Negi et al., 2020). The certification of products can be first-party, second-party, or third-party (Migliore et al., 2020; Haas et al., 2020; Xuan, 2021). First-party certification approves work from producers, while second-party certification involves certification from a group of people or a regulatory body (Sánchez et al., 2020). Finally, third-party certification is an independent certification that includes stakeholders (Bush et al., 2009; Vandergeest et al., 2015; Migliore et al., 2020). The third-party certification process creates the potential for capacity building and economic benefits to facilitate relationships among stakeholders in the sector (Bailey et al., 2016). This certification programme protects other non-target fish products (Tikina et al., 2010).

Fisheries certification allows holistic control and management of resources and processes, leading to products that are deemed to be produced according to particular standards established by the leadership and stakeholders of the certification programme (Bush et al., 2009). Independent or third-party certification of fisheries resources by Indigenous peoples is a market-based approach to the conservation and sustainable management of fisheries resources. Ensuring certification of Indigenous fisheries requires community social, economic, institutional, and cultural support (Vogt, 2000).

Various certification schemes and programs in the fisheries sector have significantly contributed to fisheries resource management (Gale & Haward, 2011; Pérez-Ramírez et al., 2012; Schebesta (2019)). Various parties have established these programs to promote or increase sustainable fisheries. Since the last several years, and notably in the last two or three, their number and the volume of certified products have risen quickly. The Marine Aquarium Council, Naturland, Marine Eco-Label of Japan, Krav, the Maritime Stewardship Council, Friend of the Sea, dolphin “friendly/safe” tuna, and the UK’s Sea fish Responsible Fishing Programme are among the programs examined. The advantages of certification programs for suppliers and other companies in the supply chain are directly related to concerns of consumer demand. When presented with two samples of the same species, such as two samples of salmon, one with an eco-label and the other without, studies of consumer reactions to seafood eco-labels frequently evaluate consumer choices (Johnston et al., 2001). According to the results, consumers favor eco-labelled products as long as the price premium is insignificant. Using a variety of fresh and processed goods, Jaffrey et al. (2001) looked at customer preferences for eco-labelling in the UK and Denmark. Once more, shoppers tended to favor labelled products over unlabelled ones. When customers were given the option between eco-labelled and noneco-labelled items of the same species, demand for eco-labelled seafood was shown to exist in both the US and Norway. However, Norwegian consumers exhibited greater price sensitivity than US consumers did. Customers are reportedly unwilling to choose a less-favorite species (i.e., to sacrifice taste) based solely on the presence of an eco-label. Even though they consider overfishing sufficiently essential, they consider changing the fish species they purchase (Johnston & Roheim, 2006). To date, indigenous communities have not realized these schemes and have not considered the traditional values, principles, knowledge, and rights of Indigenous communities in their policies (Gale & Haward, 2011). The following section considers the research approach and methods adopted for this research.

## Research Approach and Methods

### The Boundary Work Approach

This research followed a boundary work approach with our partner (Zurba & Berkes, 2014; Zurba et al., 2019a; Zurba et al., 2019b; Woodgate et al., 2017), the Mi’kmaq Conservation Group (MCG), which is a Mi’kmaq-based conservation organization for and by the Mi’kmaq people. They are under the Confederacy of Mainland Mi’kmaq (CMM). Some of the goals of this group are to manage aquatic resources and restore environmental habitats through sustainable resource exploitation (Gauthier, 2011) in tandem with the overall goal of the Indigenous certification programme. The MCG guides this procedure, resulting from their belief in Mi’kmaq spirituality and communal ideals. Apart from this profile, the MCG works with the eight mainland Mi’kmaq communities to promote education and research (MCG, 2018; Gauthier, 2011). Part of their motive is to promote and restore the resources concept and community-based activities of the Netukulimk “Take what is needed and waste nothing” way of life. This motive also conforms to the broad mission of the CMM to promote Mi’kmaq’s agenda toward self-accomplishment and enhancement of the communities (MCG, 2018).

Boundary work is a process that enables researchers to analyse boundaries, transforming co-led research into actions producing boundary objects (Zurba et al., 2019a; Drawson et al., 2017). We adopt a boundary-based work approach of collaboratively developing a policy document in the context that aligns with the review of this study as a boundary object with the MCG. This merger allowed the sharing of knowledge, information, and values by identifying the domains (problems), practice (experience and data), and the relationship generated from the collaborative initiatives (Koehrsen, 2017: Swedlow, 2017; Zurba et al., 2019a). The boundary work approach began during the shaping and scoping of the research question. We worked with the MCG to identify policy knowledge gaps and research priorities aligned with developing a Mi’kmaq lobster fishery certification. The work produced a boundary object (e.g., policy review) enabling the MCG to share information about certification with mainland Mi’kmaq communities (Zurba et al., 2019a).

Furthermore, the study also incorporated the concept of two-eyed seeing (a concept that creates, mobilizes, and translates knowledge through decolonizing Western understanding to having a common ground for co-learning and existence) (Bartlett et al., 2012). Moreover, it synergistically created knowledge from Indigenous and Western views (Peltier, 2018; MacRitchie, 2018; Elegbede et al., 2023d). We achieved this by combining the scientific and Indigenous knowledge systems highlighted earlier into the policy review and eventual synthesis of findings relating to certification schemes and Mi’kmaq values. We held monthly meetings over six months, from September 2018 to July 2019, at the MCG office, and some sessions were held physically in Truro (Mi’kma’ki/Nova Scotia, Canada). Other nonphysical meetings were held online and through telephone conversations (e.g., Skype and phone calls).

### Policy Research

Policy research methods allow us to understand policy-making processes and their effects (Kern et al., 2019). According to Browne et al. (2019), policy studies can be traditional, mainstream, and interpretive. This research adopts an interpretive policy framework that focuses on the meanings and construction of policy issues, including assumptions affecting the problems and the data used for analysis linking literature reviews, narratives, or ethnographic methods (Bullock et al., 2021; Davis & Ramírez-Andreotta, 2021). Policy analysis follows procedures such as recognizing and defining issues to be approached, identifying evaluation criteria, and adopting and evaluating alternative policies to draft the best suitable implementation strategies (Patton & Sawicki, 1993; McGregor, 2018).

#### Data gathering

**Fig. 1:**
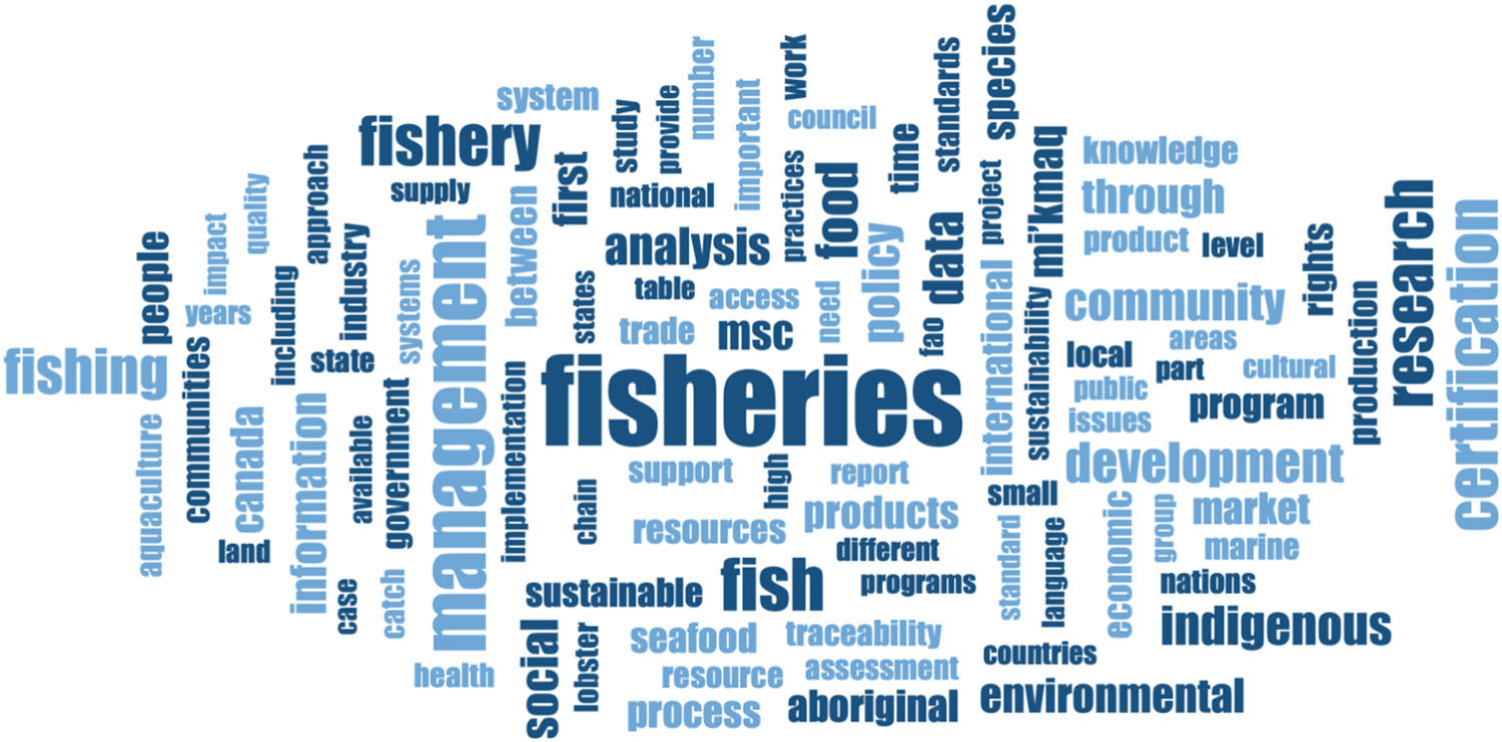
Word cloud for Mi’kmaq lobster fishery certification research

We adopted an open publication approach using search engines and indexes such as Google Scholar, Web of Science, and Scopus as described in (George et al., 2015) with main keywords such as “Indigenous,” “Certifications,” “Fisheries,” and “Mi’kmaq” (Appendix 1). These vocabularies were sorted with Boolean operators of “AND, OR” for the searches (Carlson &Palmer, 2016). We further extend our searches from other resource management sectors, such as forestry and ecotourism, to learn from the approaches used in different areas. After the investigations, approximately 8981 documents were retrieved because of the topic’s novelty and research investigation. In contrast, only a few materials were finally valuable to the context of the study. The non relevant articles were excluded by titles and abstracts and reading the entire article to ensure that some vital information could be helpful. Hence, only 267 documents were finally used and referenced in this article. The publications used for this study were structured in English and met the following criteria: containing enough evidence on Indigenous certification and relevance to the fisheries context. Following anecdotal research, these survey codes are assigned to the various keywords generated from the study. A word cloud was derived from the items reviewed, showing the frequency of essential words (Fig. 1).

The most relevant words, such as fisheries, management, certifications, Indigenous, communities, aboriginal, and fish, were generated from the literature review.

### Data Analysis and Interpretation

The analysis was performed using MaXQDa Software (MAX Qualitative Data Analysis, VERBI GmbH, Berlin, Germany). The software is highly beneficial for systematic qualitative research, which assists in developing themes from textual data (Malets, 2015; Gider & Hamm, 2019). We used this software to analyse data generated from the textual analysis and then transformed and coordinated them into various codes to form frameworks known as trees. We further identified emerging issues discussed through our boundary work stage. The codes were segmented into broad themes and subthemes through a top-down logical analysis (Kahmann et al., 2015).

The software adopted for evaluation is similar to that of Malets (2015); in their study, they used MaXQDa software to arrange data derived from documents to evaluate the relationships between local regulations and the chain of custody compliance assessment, which affect independent environmental certifications and labels. Preliminary results from their work showed that Russian local laws influenced certified forest companies due to a mismatch in domestic and international regulations and competitive price factors. The coding allows for the collection of similar textual information with the same labels and characters, thereby using the set of codes to show their relationships. There were issues in connecting some themes; however, these were later linked through subthemes. The following section considers the outcomes of this policy research, which are then grouped into the jurisdictional legal framework that supports an Indigenous -based certification programme—then outlining and elaborating on the benefits, opportunities, gaps, and challenges of promoting and participating in the Indigenous-based certification programme.

## Results and Discussion

We mapped the findings of this study into the following categories: international regulations and frameworks, national treaties and agreements that directly or indirectly affect Mi’kmaq lobster fishery certification (Appendix 2, 3), opportunities and gaps and challenges. These findings form the study’s outcome and are presented, discussed, and analysed according to how they affect Mi’kmaq lobster fishery certification.

### International Regulations and Frameworks that Affect Indigenous Communities for Certification

These provisions address the rights and interests of the Indigenous peoples, including the protection of their health, property, and heritage and the elimination of discrimination, determination, and dominance of their resources, which should consequently promote their political influence on social, economic, and cultural developments (Appendix 2). Nevertheless, some of these provisions address the right of Indigenous peoples to manage their resources, particularly fish resources and territories (Hanna & Vanclay, 2013; Washington & Ababouch, 2011; Winter, 2009). Some of the scopes of these policies, such as UNCED and UNCLOS (Appendix 2), would support and provide background for Mi’kmaq lobster fishery certification to exist and operate, particularly with its interest in the communal commercial fishery. Notably, Canadian lobster products are always in high demand in the global market (Pereira & Josupeit, 2017). With positive factors such as price, quality, and sustainability involved with Canadian lobster products, international stakeholders would likely be willing to support the Mi’kmaq by appreciating Indigenous values and identities. The CBD recognizes Indigenous communities in helping biodiversity and traditional knowledge.

Moreover, the UNCED mainly allows the labelling of environmental products; lobster is one of the products that gives consumers the allowance of choices through a market-based process—especially providing strength to Mi’kmaq lobster certification to help Mi’kmaw communities. The FAO Guidelines for Eco-labelling of Fish and Fishery Products from Marine (Inland) Capture Fisheries (FAO, 2009) call for transparent, nonstate, and market-driven certification principles. Adherence to the procedural aspect of fisheries management is one of the international provisions that would guide the certification programme as a backup (Washington & Ababouch, 2011).

### National Regulations Affecting Indigenous Peoples in Participating in Mi’kmaw Lobster Fisheries

Some provisions (Appendix 3) that support Mi’kmaq lobster certification at the national and Indigenous levels include the Aboriginal Fisheries Strategy, Aboriginal Communal Fishing Licences Regulations, and the DFO Canada’s Sustainable Fisheries Framework. Others are the Guidance on Implementation of the Policy on Managing Bycatch, the Integrated Aboriginal Policy Framework, Integrated Fisheries Management Plans, Aboriginal Communal Fishing Licences Regulations, the Supreme Court decisions of Simon, Sparrow, and Marshall, and the Modernized Fisheries Act. These policies are imperative fixtures that would constitute the components and value of Mi’kmaq lobster fishery certification. For example, the Guidance on Implementing the Policy on Managing Bycatch understands the role of Mi’kmaw Traditional Knowledge in fisheries management. This knowledge would be highly instrumental for the certification programme because of its unique historical accumulation of experience, facts, and information that would help manage the fisheries.

Since the state is in absolute control of fishing rights, full access should be extended to the Mi’kmaq to explore its resources. Hence, the Aboriginal Communal Fishing Licences Regulations further expand on the allowance of commercial fishing and FSC fishing for the Mi’kmaw people, which would support the Mi’kmaw lobster fisheries. Some of these regulations built on the Supreme Court of 1986: the Simon decision of 1986, the Sparrow decision of 1990, and the Marshall decision of 1999 (Castañeda et al., 2020). They granted the Mi’kmaq the right to engage in fishing for subsistence and commercial purposes, for example, exploration of lobster fishery resources for communal commercial benefits. The Mi’kmaq lobster certification would consider sustainability and conservation as one of its key operations goals to manage the lobster fishery. This ability allows incentives and premiums to help Mi’kmaw communities at large.

### Opportunities

#### Increased control over fisheries management and environmental protection

Certification is confirmed to improve transparency in resource governance and management (Vázquez-Rowe et al., 2016; Gulbrandsen, 2018). The Mi’kmaq lobster fishery certification would include transparency across the value chain to implement the sustainable lobster fishery by recognizing stewardship and adequate vigilance of the resources. Mi’kmaq has historical practices and traditional fishery resource management (McMillan & Prosper, 2016). Hence, Mi’kmaq leadership can support the continuous management and governance of funds and should play a leading role in decision-making. This precedence can be found in other similar use cases, according to DFO (2019), the Indigenous Broughton Clam group of the Broughton with the Archipelago of the Northern Vancouver Island of British Columbia of Canada. Both affirm the interest in controlling their natural endowments, particularly in adopting traditional management principles and safeguarding their territories and resources (Young et al., 2019; Millin, 2020). They have a historical legacy of effectively managing their resources that led to comanaged decision-making to maintain the stock of the clam fisheries (Trigona-Harany, 2017).

Traditional knowledge has the potential to promote effective fisheries management. The Mi’kmaq have accrued responsible and conservative methods of fish resource management, including ensuring the protection of the ecosystem and the surrounding environment (Thornton & Scheer, 2012; Hangle, 2018; Wheeler, Root‐Bernstein (2020)). Harris and Millerd (2010) noted that Indigenous peoples are significant in addressing climate change issues, mainly how they affect fisheries. Canada has one of the most extensive coastlines in the world, with various Indigenous peoples directly or indirectly depending on the coast and its resources (Castañeda et al., 2020). Hence, they are highly vulnerable to climate change (Sniderman & Shedletzky, 2014). Mi’kmaq lobster certification could recognize fish stocks and factors such as ecosystem pressures and climate change issues, including natural and anthropogenic disasters (Borland, 2016), by adopting third-party non-fishery-specific environmental certification schemes, such as the European Eco-Management and Audit Scheme (EMAS), ISEAL, and ISO.

#### Social benefits and capacity building for the Mi’kmaw communities

The Mi’kmaq lobster certification can serve as a platform to attract benefits such as capacity recognition and building for the Mi’kmaq. One way to explore these benefits could be through the premium consumers pay for lobster products. The price premium is an additional price placed on the fish product due to its value and the demanding financial process of the sustainability standards and certification programme that the product passes through (Bailey et al., 2016). This price is required because it justifies and offsets the cost of instruments, tools, and independent capacities prior to labelling (Roheim et al., 2011; Furumo et al., 2020). However, as the price generates value, Mi’kmaq communities can support and independently fund their social and cultural activities, such as schools and recreational community centres. The economic value from the proceeds needs to further help with practical training and capacities that will equip Mi’kmaw personnel and guards on fisheries management activities.

The Mi’kmaq can harness positive impacts on social benefits through the participation of stakeholders. A well-drafted Mi’kmaq lobster certification scheme can be fully utilized to emancipate the Mi’kmaq, including other stakeholders, in many ways. The capacities of the Mi’kmaw communities are promoted through continuous training and capabilities, thereby revalidating the promotion of environmental and resource management with positive activities and practices. Certification has dramatically allowed non-Indigenous managers to consult with the community (Washington et al., 2011). This was the case for the following certifications for the forestry sector in Canada: The Forest Stewardship Council (FSC), the Canadian Standards Association (CSA), and the Sustainable Forestry Initiative (SFI). In addition, some certification is linked with enhancing working conditions, learning and knowledge sharing, gender empowerment, and social capital systems that facilitate collective decision-making (George et al., 2022; Van der Ven et al., 2018). The few social advantages of MSC certification need to be recognized (Carlson & Palmer, 2016).

#### Potential for networks with stakeholders to harness market access through fish product differentiation and promotion

The Mi’kmaw Lobster Certification can improve relationships between stakeholders and the Mi’kmaq people. Collier et al. (2002) argue that FSC certification can improve relationships between stakeholders because of the duty to consult with local communities (including Indigenous communities) imposed on non-Indigenous certified companies. Again, this is not a concept the Mi’kmaq could impose externally through their certification scheme or the certification of their products. However, Mi’kmaq has practical co-management skills on fishery resources with various stakeholders, such as NGOs, academic institutions, and governmental institutions (Fox, 2006; Chute & Speck, 1999; Crook et al., 2016). This initiative would further allow support to adequately manage the lobster fishery by harnessing the opportunities from certifications (Tikina et al., 2010).

The stakeholder relationship could facilitate adequate market access, thus promoting the chain of custody certification to bring a decisive advantage for consumers’ preference for the certified product and label, including structures for the certified product (Tikina et al., 2010). It is not worth noting that access to the market is one factor in adopting NGO certification schemes. This assertion is reflected in the FSC and MSC programmes, which have made certification a platform for integrating fish products into possible markets. In addition, the impact of accreditation in penetrating the market and giving consumers confidence cannot be overemphasized (Carlson & Palmer, 2016). Additionally, all commercial lobster catches in Canada are already certified by MSC certification. This situation could also include communal commercial licences. However, the MSC programme has yet to recognize the Miqmaw leadership in the certification process, mainly to formally show the label as an Indigenous recognized. It remains unclear whether the MSC is a privately owned label that does not recognize Indigenous social attributes in its criteria and principles.

The market for lobster is local and external; however, penetrating external markets through exporting the certified lobster could justify initiating and developing the Indigenous certification programme by the Mi’kmaq. Moreover, Carlson and Palmer (2016) confirmed that FSC certification supports producers with market access for exportation while maintaining customer ties. Lobster certification would enable actors to adhere strictly to traditional sustainable practices for proper cultural, economic, and social performance. Furthermore, consumers and the broader market could differentiate themselves from other lobster labels. This situation would help explore price premiums for adequate market penetrations, provided that the consumer is willing to pay a premium for Indigenous-certified lobsters, thus developing financial support for the Mi’kmaq to fund and maintain the certification programme.

#### Sustainable approaches of community-owned commercial fisheries for communal benefit

Community-based commercial fisheries promote biodiversity for the Mi’kmaq in a way that helps to adopt relevant fisheries management practices, thus encouraging collaborative opportunities (Thornton & Scheer, 2012). For example, Guam’s Indigenous fishing community, through Guam Fishermen’s Cooperative Association (GFCA), developed the best method of the sustainable fishery to connect cultural fisheries strategies in the state’s fisheries framework (Weijerman et al., 2016; Richmond & Kotowicz, 2015), thus developing safety programs for fishing with the inclination of their cultural practices. They support their community fishers to consider the deepwater fishery to replace inshore fishing to modify their economic and cultural identities (Allen & Bartram, 2008).

Mik’waw nations engage in a community-based administration programme for its fisheries. This engagement has helped in the development of the leadership and governance structures of the community (Wiber & Milley, 2007). According to Milley & Charles (2001), Mi’kmaq adopts a community-based approach with the traditional principles of Netukulimk to attain dominance in fisheries management. Thus, they are graduating indigenous-based fisheries experts for conservation and fisheries management policies (Milley & Charles, 2001; McMillan & Prosper, 2016; Castleden et al., 2017). The proposed Mi’kmaq lobster certification would significantly include a community-based approach to uphold mutual interest.

The Mi’kmaq lobster certification would enable the communities to benefit on a collective communal basis. It will be in terms of independence and sovereignty to upgrade the status of the Mi’kmaw communities. The indigenous communities of the Peruvian Amazon could explore commercial sustainable forest management to support their community after they had won the right to their territories in 2002 (Horn et al., 2012; Blackman et al., 2017). This achievement was a success through their Traditional and Indigenous Knowledge with their developmental plan called the Planes de Vida (life plans). In addition, FSC principles helped improve production and sustainably accrue communal benefits in exploiting rubber resources (Francesconi et al., 2018). This integration enables people to earn approximately 80 percent of their income from sustainable timber production (Zwick, 2018). The local and international provisions could empower Mi’kmaq to engage in commercial fishing for a better livelihood for Mi’kmaw communities.

#### Augment regulations with political relevance

Mi’kmaq lobster certification could strengthen regulations. These possibilities are essential when there are uncertainties regarding agreement issues between the Indigenous group and the government (Tikina et al., 2010). Certification has helped fishers utilize fisheries exploitation allocation and concession, including legally endorsing their resource rights and tenure (Lallemand et al., 2016). In addition to augmenting government regulations, there are chances of governmental support through waivers and reliefs of benefits such as tax supply of capacities, incentives, and further support for the Indigenous peoples in allocating their resources and rights. For instance, Bolivian FSC forestry certification successes in continuous transparency operations, including acceptable management practices, prompted the government to exempt some levels of taxes (Espinoza & Dockry, 2014). This circumstance facilitates fisheries-supported projects and infrastructures, such as improved road access, electricity, and enhanced fish processing structures and plants (Carlson & Palmer, 2016). Mi’kmaq lobster certification should conform to local- and international-level regulations.

#### Enhancement and value addition to the seafood product

The Mi’kmaq lobster certification programme would add value to the lobster fish product. For example, the Indigenous People of Manjimup, Grafton, and Hobart in Australia contributed significantly to their forest timber and wood products through processing, including downstream value addition (BDO consulting (2004); Gavran et al., 2012). This certified wood produces more economic value for Indigenous peoples, their ability to use resources, and a keen interest in sustainability (Miyata, 2007; Saifullah et al., 2018; Elegbede et al., 2023a; Elegbede, 2021; Akintola et al., 2021).

It has been confirmed by Brown et al. (2016) that certification of seafood products improves the value of the product and the fisheries by enhancing responsible and credible practices, primarily with the participation of fishers at the local level. The product amount further promotes market accessibility and penetration, especially at the post harvesting stage, and is invariably more relevant than direct price premiums (Elegbede et al., 2021; Foley, 2012). This consideration has caused Indigenous -based certification to promote socioeconomic well-being and livelihood, recognized in the corporate social responsibility (CSR) context. This factor is at the core interest of the consumer to support the certification programme, hence adding substantial value to the uniqueness of the certified product (Del Giudice et al., 2018). In addition, the high value attributed to the certified lobster product—the characteristics of the Mi’kmaw organizations and philosophies, worldview concerning sustainability, and a keen passion for protecting Mother Earth and its resources for future generations—would add more value to the lobster product. The lobster product from this exercise would be promoted and supported to give additional cost and mechanisms with sustainability principles (Elegbede et al., 2023b). However, this situation might prompt excessive and illegal fishing practices, but indigenous traditions are known as traditional sustainability practices.

#### Adoption of Mi’kmaw ecological knowledge (MEK) as a basis for fisheries management

Mi’kmaq Ecological Knowledge (MEK) (Kwilmu’kw Maw-klusuaqn Mi’kmaq Rights Initiative (2007)), which was pronounced by CNN as the basis for the development of an Indigenous certification scheme, would be fundamentally important in the Mi’kmaq lobster certification programme. Knowledge such as MEK is a subset of traditional knowledge organized by Indigenous communities on the relationships between the natural environment and the people (Finn et al., 2017). MEK is a holistic or comprehensive collection of the knowledge that Mi’kwaq possesses based on their close relationship with their natural habitats, which involves exploitation, conservation, and spiritual ideologies and has passed on based on generation to generation, “kisaku kinutemuatel mijuijij” elders to the child" (Chambers, 2019; Warrior, 2020). This knowledge is bounded by Mist’no’kmaq (Guidance toward sustainability practices) and Netukulimk (Mi’kmaq’s sustainability principles on collaborative and generational exploitation of resources) (McMillan & Prosper, 2016; Chambers, 2019; Warrior, 2020).

In Canada, Indigenous knowledge has helped to facilitate certification in the aquaculture industry. For example, Ahousaht First Nation, founded on Flores Island in Clayoquot Sound of British Columbia, engages in salmon fish farming. Their fish was certified by the CERMAQ, the first Canadian third-party certification for fish farming and feed, to promote sustainable aquaculture focusing on sustainability principles and independent auditing (Wewerinke-Singh & Hamman, 2020; Assembly of First Nations, 2011; Harris et al., 2001). This programme worked with stakeholders to enhance sustainable fish farming and its associated activities for protecting and improving the environment (Elegbede et al., 2022).

Intrinsically, the CERMAQ completed a pilot project for an Indigenous certification programme in 2011 with the mainstream and Ahousaht First Nation after successful auditing and accreditation towards achieving Indigenous certification on the Aboriginal Principles for Sustainable Aquaculture (APSA), which considers sustainability principles with auditing standards. The accreditation of the CERMAQ for fish farming operations yielded opportunities such as creating new sites, operational support, and employing local personnel (Assembly of First Nations, 2011; Harris et al., 2001).

The applied systems of knowing known as IK systems are founded on thousands of years of observations. With repeated observations made across a range of time scales, these knowledge systems can offer a level of integrity that is calibrated. The methods used for knowledge storage, transmission, and application are where Indigenous and science-based knowledge systems diverge (Brosius, 2006). The Indigenous knowledge systems are connections between the ecological context in which they function and the cultural paradigm from which they emerged (Brosius, 2006). The Indigenous people are the keepers and practitioners of the expressions of knowledge that are influenced by their beliefs, spirituality, and cosmology and directed by the knowledge of their predecessors (Bruchac, 2007). Indigenous people converse with their peers in social situations to discuss their experiences, drawing on their shared knowledge and language. Each town has its subject matter experts for various occupations. These specialists’ collective knowledge makes it easier to integrate their insights into a more comprehensive frame of reference for their ecological surroundings. Fox (2006) affirms that local knowledge from the Mi’kmaq is pertinent to the thriving commercial fishery for Mi’kmaw communities. In another report, the Lobster Management Plan (LMP) designed by Mi’kmaq was a success for lobster fisheries because it introduced Mi’kmaq Ecological Knowledge (MEK) to support sustainable resource harvesting at the local level (Huber, 2009).

### Gaps and Challenges

#### Translation of traditional knowledge by Indigenous peoples into technical indicators

There could be difficulties in integrating Traditional knowledge with conventional indicators. This issue is essential because of the need to adhere to international regulations and standards, such as the International Organization for Standardization (ISO). In the forest industry, the FSC and the Canadian Standards Association and Sustainable Forest Management (CSA-SFM) recognize the importance of Indigenous values for sustainable forest management (Clark & Kozar, 2011). Thus, the use of traditional knowledge in management and planning is emphasized. They further stress the need to consider adequate consultation and adoption of this knowledge in developing the criteria and indicators, including values and goals for sustainability certification (Smith, 1998). It has been challenging to integrate TEK into the sustainability certifications programme (Khalid et al., 2021).

The Indigenous resource Alliance fishers from the Kitasoo/Xai’xais, Heiltsuk, Nuxalk, and Wuikinuxv First Nations of BC. In partnership with Ban and her collaborators, BC’s Central Coast has experience in rockfish (Sebastes spp.) management with a massive demand for fisheries (McGreer & Frid, 2017). Management included traditional and local ecological knowledge, which provided the foundation to quickly understand the change in fish populations and take appropriate actions (McGreer & Frid, 2017). To the Mi’kmaq, the traditional understanding of resource management is a holistic way of life that can improve resource management because of the historical knowledge gained over the years (Parsons & Prest, 2003). For this knowledge to be helpful for lobster certification, incorporating the Two-Eyed Seeing principles in the certification programme would be essential. Two-eyed seeing has been a successful approach to capacity building and education in Atlantic Canada (McMillan & Prosper, 2016). The Mi’kmaq lobster certification, through its leadership, can use the concept as a guiding principle for integrating the most appropriate Western knowledge with Indigenous background through collaborative and interdisciplinary engagement (Zurba et al., 2023; McMillan & Prosper, 2016).

#### Cost of certifications and financing

Certification cost is a crucial factor to consider for Mi’kmaq lobster fishery certification. Apart from recognizing other sustainability variables, adequate financing of the operations of a certification scheme allows the smooth running and practical application of its principle (Roheim et al., 2018; Van Putten et al., 2020; Stawitz et al., 2017). A continuous fund would be required to offset the third-party certification and auditing process that follows international regulations and rules (Roheim et al., 2011). However, with the price premium, it might be easier to generate sufficient profit to cover the cost of certification, evaluation, and audit processes (Klooster, 2006).

It is essential to look for ways that Mi’kmaw fishers can benefit from the Mi’kmaq lobster certification programme by finding ways to reduce the cost of participation. The financial resources to facilitate the application could be gathered through a price premium of products and government or nongovernmental support. It is on record that the Federal First Nation forestry programme supported the eel fisheries for funding (Reference?). Additionally, the Federal Indian and Northern Affairs of Canada and the First Nation forestry programme have helped the forest operation services (Reference?). Furthermore, NGOs such as Ecotrust Canada supported the Tsleil-Waututh First Nation (Tikina et al., 2010). With Wahkohtowin’s Indigenous Guardian Program, for instance, young people from First Nations communities in NES can reconnect with their ancestral lands and waters. The program encourages young people to engage with older people and learn about traditional knowledge while preparing them for professions in local businesses and the management of natural resources. By providing First Nation housing requirements with locally sourced and treated wood, Ecotrust Canada supported the “Tree to House initiative” by Wahkohtowin (Drawson et al., 2017). The United States government has accountable support for its forest certification programme. This support is considered a trust responsibility to the Indigenous nations of the US and is mainly in the form of subsidies or payments for ecosystem services (Tikina et al., 2010).

#### Chain of custody and its effect on the price premium

The chain of custody affects the high price of fish products. Price premiums have been considered a critical indicator for examining the effectiveness of certification by serving as the central platform for providing market-based incentives for fisheries (Roheim et al., 2011). For example, the average cost for MSC certification ranges between US$10,000 and US$ 500,000 for small-scale and large-scale fisheries. These costs are used to support the additional cost of certification, which is part of the maintenance cost for a sustainable fishery (Roheim et al., 2011; Van Putten et al., 2020; Elegbede, 2023c).

The chain of custody also ensures transparency in developing lobster products for consumers. This positivity is reflected in the price premium attached to the product, used to improve the value and reputation of the standards, thus differentiating it from other measures (Bartley, 2010; Asche & Bronnmann, 2017; Gilani et al., 2016) despite producers not actively benefitting from price premium in the long run. This observation lies in a study conducted by the University of Rhode Island for price premiums at the retail level in the fishery. Apart from producers, consumers would want to pay for the products. However, retailers benefit more because the producers rely much on the retailers who are not ready to pay a price premium to suppliers. Hence, they control market accessibility for the product (Carlson & Palmer, 2016). The lessons learned from the various ventures show that appropriate measures to reduce the load and burden of lobster prices can decrease costs incurred in the chain of custody processes. This approach would help to implement the Mi’kmaq lobster certification programme properly.

#### Perception of Indigenous-based criteria and indicators

Criteria and indicators from the Mi’kmaw communities for the sustainability of natural resources are highly recognized by the local communities (Nova Scotia) and environmental certification schemes. Measures and indicators are critical drivers of certification standards, and criteria are essential tools to achieve an initiative or goal. At the same time, markers, gene names, or symbols are the directions to maintain and assess these criteria (Sherry et al., 2005). Traditional indicators derived from Mi’kmaw values and identities are more encompassing than conventional ones. A comparison between the FSC-boreal and an Indigenous land stewardship indicator shows that the Mi’kmaw communities have detailed indicators that are better than the FSC in areas such as the inclusion of resource access, respect, and dignity of their status in the resource chain of custody (Tikina et al., 2010).

The Mi’kmaq lobster certification could incorporate criteria and Indicators produced and promoted by Mi’kmaw communities. Measures and indicators that are indigenous-based are valuable tools for lobster resource management and conservation because it would be a bottom-up approach where communities and other stakeholders can contribute and participate in the decision-making process.

A further comparison of locally staged frameworks of Indigenous and non-Indigenous criteria, including indicators, determines how local structure differs from the other top-down frameworks. The principles and symbols used by the Tl’azt’en First Nation retrieved from their archives of communal information for sustainable forest management were used to compare the other frameworks, such as the Canadian Council of Forest Ministers’ (CCFM), the Local Unit Criteria and Indicators Development (LUCID) and the Centre for International Forestry Research (CIFOR) frameworks (Sherry et al., 2005). The First Nation communities developed their criteria and indicators from its members on sustainable forest management with more than 100 interviews based on a grounded theory content analysis (Sherry et al., 2005). This initiative allows Indigenous communities to express their local knowledge, practices, and beliefs to manage their resources effectively. Thus, allowing the Indigenous criteria to prevail through a bottom-up approach would require frequent appraisal and changes because of local priority shifts.

#### Capacities of the Mi’kmaw over sustainability and certification

Due to insufficient capacity, Mi’kmaq lobster certification might face unsteady quantities and qualities of lobster fish products. Hence, this further affects the continuous supply and access to the market (Carlson & Palmer, 2016). The Aboriginal Fisheries Guardian program (AFGP) has been designed to objectively upgrade the insufficient capacities of Indigenous peoples as guardians to manage their fishery resources. These guardians observe, record, and report violations of the Fisheries Act and help with projects and community involvement/education. Therefore, collecting and managing relevant information for proper monitoring and surveillance could play a key role in enforcing the legal justification raised in the Fisheries Act. Applying this to Mi’kmaq lobster certification would allow Guardians to be actively involved in ensuring overall fisheries management and improving the sustainability and stewardship of fisheries (Trant et al., 2012; Orton, 1995; Durette, 2018).

According to Bennett et al. (2018), the availability of Indigenous-based guardians on the coast would allow adequate law enforcement, monitoring of environmental parameters, and support of stewardship efforts. However, fish stock management could be improved if the capacities of the Mi’kmaw communities and organizations were adequately explored.

#### Barriers to market entry due to human rights issues

In general, seafood is faced with barriers to market entry. Unlike the other areas, this market barrier is caused by the immense technological and economic resources of processing and access to fishing rights, including resource allocation leading to market barriers (Auld, 2014). These factors significantly affect small-scale fishers, particularly Indigenous fishers. The third principle of the FSC criteria recognizes and respects the rights of Indigenous peoples to promote Indigenous peoples’ rights. Furthermore, the FSC still allows the identification of the risk type in the value chain of its commodity (Gale & Haward, 2011). Canada has yet to fully adopt the UN Declaration on the Rights of Indigenous Peoples (UNDRIP) into her terms of engagement with Indigenous Peoples. However, this would not constitute a barrier for the Mi’kmaq to access their lobster markets in that both the Indigenous and nonindigenous Canadians have the right to practice traditions, customs, and livelihoods that enshrine the uniqueness of the groups before colonization (Tikina et al., 2010).

#### Influence of external and international regulations

Canada once faced a reduction in seal catches and export of this product. These challenges were due to campaigns over the kind of harvesting of the Seal product. Hence, there was a ban on harvested Seal products from the European Union (EU) in 2009. Afterward, a second-party certification from the EU gave licences to Indigenous hunters. They only considered the products of the Inuit due to their traditional way of life dependent on hunting (European Union, 2009). The socioeconomic interests of the Inuit are concerned with subsistence, a critical segment of their culture and values. This right also has the backing of the United Nations Declaration on the Rights of Indigenous Peoples (UNDRIP) (Lafrance, 2017).

Despite this interest, this consideration led to the collapse of the seal market. Based on the challenges in the industry, including the series of bans and price collapse, various external and internal interventions have been initiated, such as the World Trade Organization (WTO) challenging the EU ban on seal products. The lesson learned from the seal certification is that the Mi’kmaq-based fisheries certification should consider international regulations for its fisheries programme, creating alternative market access for the lobster fish product.

## Conclusions

This gap, challenge, and opportunity study points toward understanding the potential for Indigenous lobster fishery certification for Mi’kmaw communities, which is envisaged to promote Indigenous governance for commercial fisheries. Local, international, and national regulations support embarking on Indigenous certification. The feasibility indicates that the Mi’kmaq community is attempting to develop adequate abilities to adopt the identities of their social and cultural values toward empowering the communities in harnessing economic benefits on a communal scale. Furthermore, certifying the lobster fishery based on Mi’kmaw values would strengthen Mi’kmaw governance toward increasing control and adaptation to environmental protections. Social collective benefits and capacities promote stakeholders’ involvement in promoting access to seafood products to local and international consumers. However, there may be disagreement about integrating the traditional understanding of Indigenous peoples with the existing conventional code of conduct for responsible fishing. Correspondingly, the certification cost could affect the governance and management structures, perhaps causing market entry and financing barriers. This situation can be resolved mainly through price premium market-based systems and then influenced by external and internal regulations. Future research should evaluate the components of the certification scheme and how they could be used to support Mi’kmaq’s cultural identities and livelihood. We further evaluate how Indigenous-based certifications could create economic, social, and governance opportunities and divert funds from the industry to Indigenous communities by acquiring two of Clearwater Seafood Inc’s eight shares (Clearwater). They are North America’s most extensive shellfish harvesting, processing, and retail enterprise, also known as the single most significant investment in the entire seafood sector by an Indigenous consortium in Canada. The consortium shareholders include international corporation premium brands and FNC Holdings Ltd, a coalition of seven Mi’kmaq bands.

By exploring the realm of Indigenous certification within the lobster fishery, this study not only addresses the existing gap, challenge, and opportunity but also sheds light on broader implications that transcend beyond regional and sector-specific boundaries. The findings would be relevant with scholars working in various areas worldwide all over the world, as they touch upon fundamental themes of sovereignty, which extend far beyond the specific context of Mi’kmaw communities. The examination of commercialization and labeling practices within the Indigenous lobster fishery offers insights into broader debates surrounding the Indigenous reclamation of control over resources. This inclusion invites scholars from different disciplines to engage with the study’s findings and contribute to the wider academic conversation.

Conclusively, the study underscores the significance of Indigenous governance and its potential impact on environmental resource protections. By certifying the lobster fishery based on Mi’kmaw values, the study highlights the broader implications of such initiatives, transcending the specific region and resource sector. This facet of the research aligns with broader discussions on Indigenous sovereignty, self-determination, and the reclamation of control over natural resources, resonating with scholars working on similar themes globally.

### Supplementary Information

Appendices&Supplementary

## Footnotes

1 *Netukulimk* is considered sustainable resource management, appreciating and recognizing the past, being accountable for the present and preserving the future (Capistrano & Charles, 2012).
