# Supplementary material for: Gaps and Challenges in Harnessing the Benefits and Opportunities of Indigenous Certification for a Sustainable Communal Commercial Lobster Fishery
Source: Environ Manage. 2023 Jul 20;72(5):902–21. doi: 10.1007/s00267-023-01852-7 (PMC10509051; doi:10.1007/s00267-023-01852-7)
Supplement: Supplementary file 1 — Appendices&Supplementary [file 267_2023_1852_MOESM1_ESM.docx]

Appendices

Appendix 1. Search themes used to identify literature on the study

| Main Searched keywords | |
| --- | --- |
| Benefits AND Indigenous certification | Indigenous AND Certification |
| Opportunities AND Indigenous certification | Fisheries AND Indigenous certification |
| Gaps AND Indigenous certification | Forestry AND Indigenous certification |
| Challenges AND Indigenous certification | Indigenous AND Certification |
| Sub searched keywords | |
| Policies, regulations AND Indigenous peoples | Mi’kmaw AND stakeholders |
| Resource control and Indigenous | Market access AND fish product differentiation and promotion |
| Resource management AND Indigenous | Communal benefit AND commercial fishery production |
| Social benefit AND Mi’kmaw communities | Capacities people over the certification |
| Dominance AND certification | Marketing, right AND certification |
| Traditional knowledge and indicators | External and international regulation influence |
| Cost of certification | Licensing and certification |
| Chain of custody AND price premium | Capacities people over the certification |

Appendix 2. International policies and declarations affecting indigenous peoples and fisheries

| Year | Provisions | Description |
| --- | --- | --- |
| 1945 | Indigenous rights Charter of the United Nations | Addresses one of the demands of the Indigenous peoples on the principles of equal rights and self-determination of peoples |
| 1948 | Universal Declaration on Human Rights | Considered rights, also affecting the Indigenous people such as the right to free association, property, health, education and life |
| 1965 | UN Convention on the Elimination of All Forms of Racial Discrimination | This convention talks about eliminating racial discrimination from the traditional group such as Indigenous group |
| 1966 and applied in 1976 | International Covenant on Economic, Social and Cultural Rights | Stipulate that all peoples have rights to self- determination to openly explore their political interest for social, economic and cultural developments. |
| 1982 | United Nations Convention on the Law of the Sea (UNCLOS) | Coastal states are equipped with rights to explore and exploit, conserve and manage the living resources in the 200 nautical miles of the EEZ. This jurisdiction also relates to the Indigenous people when the states have given them the rights to control their resources |
| 1989 | Indigenous and Tribal Peoples Convention (International Labor Organization C169 | It talks about the rights and empowers Indigenous peoples to be consulted by the government in any administrative or legislative issues and allow the peoples to participate in decision making |
| 1991 | World Bank Operational Directive | The directive guides World bank on giving funds and support to borrowers working on projects that affect Indigenous peoples |
| 1992 | Declaration on the Rights of Persons Belonging to National or Ethnic Religious and Linguistic Minorities | Allows the minority to effectively uphold their freedom and human right with equity and nondiscrimination before the law. |
| 1992 | Rio Declaration on Environment and Development | Emphasize the importance of the Indigenous traditional knowledge in environmental management by the Indigenous people |
| 1992 | Convention on Biological Diversity | Acknowledge the status of the Indigenous peoples in fostering biodiversity with conventional knowledge |
| 1992 | UNCED | Agreement support labeling of environmentally supported products to support choices of consumers, through a market-based process |
| 1993 | Vienna Declaration and Programme of Action | Act for the creation of a working group on declaring the rights of Indigenous peoples and denial of self-determination are violations of human rights also giving importance to the realization of these rights |
| 1995 | FAO Code of Conduct for Responsible Fisheries (the Code) | Indicate uncertainties on management advice on precautionary methods on fisheries |
| 1995 | United Nations Fish Stocks Agreement | Agreements on the long-term conservation and sustainable use of straddling and migratory fish stocks |
| 2001 | UNESCO Universal Declaration on Cultural Diversity | Talks about the defending cultural diversity as ethical and as the same as human dignity, with emphasis to tights of Indigenous peoples as a commitment to human rights and freedoms |
| 2003 | Equator Principles | Consideration of Indigenous peoples, as stakeholders on analysis of risk on developmental projects by banks |
| 2005 | World Bank Operational Policy (OP) and Bank Procedure (BP) | On financing of projects that affect the Indigenous peoples |
| 2005 | FAO Guidelines for Eco-labeling of Fish and Fishery Products from Marine (Inland) Capture Fisheries | This guideline allows certification principles to align with concerned international regulations, and to be transparent market was driven and voluntary. Minimum requirement should be conducted under best-practiced management systems, with stocks not overfished and properly addressing the unfavorable impact of the fishery on the ecosystem. Following adequate procedural and institutional aspect of the certification scheme |
| 2007 | UN Declaration on the Rights of Indigenous Peoples (UNDRIP) | Outlined the various right of the Indigenous peoples and approved the states should give a free, prior and informed consent to the Indigenous peoples about projects that affect their livelihood |

Source: (Hanna & Vanclay, 2013; Washington & Ababouch, 2011; FAO, 2009; Winter, 2009).

Appendix 3. National and Aboriginal regulations affecting the Indigenous peoples in participating in fisheries

| Year | Provisions | Description |
| --- | --- | --- |
| 1992 | Aboriginal Fisheries Strategy | It is a response to the Supreme Court decision of Canada on proceeding guideline for fisheries management by the Indigenous peoples for food, social and ceremonials, enhancing their capacities and skills, and promoting management of its resources |
| 1993 | Aboriginal Communal Fishing Licenses Regulations | Elaborate on communal fishing licenses and its management responsibilities including the funds attached, thereby allowing both commercial fisheries and food, social and ceremonial (FSC) fisheries |
| 2007 | The Integrated Aboriginal Policy Framework | Enables the DFO to engage with the Indigenous groups with mutual and respectful relationships and their involvement in decisions making with a stewardship agreement that is consistent according to the constitutional rights |
| 2009 | DFO Canada’s Sustainable Fisheries Framework | Covers the use of precautionary methods to fisheries management with support on biological reference pints and species stock status |
| 2013 | Guidance on Implementation of the Policy on Managing Bycatch | Categories catch in policy and involvement of bycatch in management. Thereby recognizing the role of Traditional Ecological Knowledge (TEK) and Aboriginal Traditional Knowledge (ATK) in data gathering |
| 2013 | Integrated Fisheries Management Plans | Aimed at giving a framework for the fisheries resource sustainability over a specific time duration, collaboratively designed with stakeholders including Indigenous groups |
| 1993 | Aboriginal Communal Fishing Licenses Regulations | Elaborate on communal fishing licenses and its management responsibilities including the funds attached, thereby allowing both commercial fisheries and food, social and ceremonial (FSC) fisheries |
| 1986, 1990, 1999 | Supreme Court of 1986 (Simon decision: 1986), (Sparrow decision: 1990) and (Marshall decision: 1999) | The Simon decision of 1986 recognizes peace and friendship treaties with land rights with the Indigenous peoples. The Sparrow decision of 1990 allows the states to recognize the aboriginal rights to hunt and fish for food, social and ceremonial; and (Marshall decision, 1999), approved the treaty Right of the Mi’kmaw, Maliseet and Passamaquoddy peoples to depend on natural resources for modest living and benefit to the community. |
| 2019 | Modernised Fisheries Act | Empowering the Indigenous communities on fish habitat decisions, reviews and monitoring of projects and recognizing the rights, respect, partnership including cooperation with the Indigenous peoples. |

Source: (Wiber & Milley, 2007; Archibald & Rangeley, 2018; Capistrano, 2010; Department of Fisheries and Oceans, 2007; Harris & Millerd, 2010; Fisheries and Oceans Canada, 2019; ICES, 2020)
